# Predicted metabolic roles and stress responses provide insights into candidate phyla Hydrogenedentota and Sumerlaeota as members of the rare biosphere in biofilms from various environments

**DOI:** 10.1111/1758-2229.13228

**Published:** 2024-01-09

**Authors:** Emilie J. Skoog, Tanja Bosak

**Affiliations:** ^1^ Department of Earth, Atmospheric and Planetary Sciences Massachusetts Institute of Technology Cambridge Massachusetts USA; ^2^ Integrative Oceanography Division Scripps Institution of Oceanography, UC San Diego La Jolla California USA

## Abstract

Pustular mats from Shark Bay, Western Australia, host complex microbial communities bound within an organic matrix. These mats harbour many poorly characterized organisms with low relative abundances (<1%), such as candidate phyla Hydrogenedentota and Sumerlaeota. Here, we aim to constrain the metabolism and physiology of these candidate phyla by analyzing two representative metagenome‐assembled genomes (MAGs) from a pustular mat. Metabolic reconstructions of these MAGs suggest facultatively anaerobic, chemoorganotrophic lifestyles of both organisms and predict that both MAGs can metabolize a diversity of carbohydrate substrates. Ca. Sumerlaeota possesses genes involved in degrading chitin, cellulose and other polysaccharides, while Ca. Hydrogenedentota can metabolize cellulose derivatives in addition to glycerol, fatty acids and phosphonates. Both Ca. phyla can respond to nitrosative stress and participate in nitrogen metabolism. Metabolic comparisons of MAGs from Shark Bay and those from various polyextreme environments (i.e., hot springs, hydrothermal vents, subsurface waters, anaerobic digesters, etc.) reveal similar metabolic capabilities and adaptations to hypersalinity, oxidative stress, antibiotics, UV radiation, nitrosative stress, heavy metal toxicity and life in surface‐attached communities. These adaptations and capabilities may account for the widespread nature of these organisms and their contributions to biofilm communities in a range of extreme surface and subsurface environments.

## Graphical abstract

Pustular mats from Shark Bay, Western Australia, host diverse microbial communities within an organic matrix. We analyze metagenome‐assembled genomes (MAGs) of two rare biosphere members: Candidate phyla Hydrogenedentota and Sumerlaeota. Metabolic reconstructions of these MAGs reveal facultatively anaerobic lifestyles, diverse carbohydrate metabolisms, and multiple adaptations and stress responses which likely contribute to their prevalence and roles in biofilm communities across varied extreme environments.

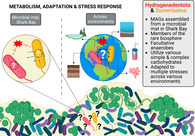

## INTRODUCTION

Pustular microbial mats from the hypersaline waters of Shark Bay, Western Australia, are some of the best‐known modern analogs of Proterozoic peritidal mats (Golubic & Hofmann, 1976; Hofmann, 1976; Jahnert & Collins, 2013; Logan et al., 1974; Moore et al., 2020; Moore et al., 2023). Key microbial groups within this system have been identified as Proteobacteria (~40%), Bacteroidetes (13%–24%), Planctomycetes (~13%), Chloroflexi (3%–12%), Verrucomicrobia (2%–10%), Cyanobacteria (~4%), and other less abundant phyla (i.e., Acidobacteria, Spirochaetes, GN04, Gemmatimonadetes, Actinobacteria, Myxococcota) (Allen et al., 2009; Babilonia et al., 2018; Cutts et al., 2022; Goh et al., 2009; Ruvindy et al., 2016; Skoog et al., 2022; Wong et al., 2015; Wong et al., 2018; Wong et al., 2020). The inferred relative abundances of these microbes are based on both 16S rRNA amplicon and metagenomic analyses (Skoog et al., 2022; Wong et al., 2015). Uncharacterized candidate bacterial phyla occur at <1% abundances but make up to ~10% of the community (Ruvindy et al., 2016; Skoog et al., 2022; Wong et al., 2015). Among these, Candidate phyla Hydrogenedentota (previously NKB19) and Sumerlaeota (previously BRC1) are repeatedly reported within pustular mats in Shark Bay (Babilonia et al., 2018; Skoog et al., 2022; Wong et al., 2015), yet their roles and metabolic capabilities remain poorly understood.

Previous 16S rRNA amplicon sequencing (Wong et al., 2015) and metagenome‐assembled genome (MAG) analyses (Babilonia et al., 2018; Skoog et al., 2022) identified Candidate phylum Hydrogenedentota in Shark Bay. Additional 16S rRNA amplicon sequencing analyses revealed the presence of Hydrogenedentota in microbial mats from the Great Sippewissett salt marsh, Falmouth, MA (Armitage et al., 2012) and Guerrero Negro (GN), Baja California Sur, Mexico (Kirk Harris et al., 2013), Tibetan hot springs (Zhang et al., 2018), the Bohai Sea, Yellow Sea, and South China Sea (Zhang et al., 2019), in sludge from anaerobic digesters (Rivière et al., 2009), and in Laguna Llamara and Laguna Cejar salt flat lagoons in the Atacama Desert, Chile (Rasuk et al., 2016). Studies that reported abundances found that organisms of this candidate phylum always constituted <1% of the community. Currently, there is only one Hydrogenedentota MAG available from Shark Bay (Skoog et al., 2022), but MAGs of Hydrogenedentota have been assembled from other environments including subsurface waters (Momper et al., 2018; Mosley et al., 2022; Tully et al., 2018), hot spring water and sediments (Liew et al., 2022; Zhou et al., 2020), marine sediment (Zhou, Mara, et al., 2022), freshwater sediment (Hahn et al., 2022; Jaffe et al., 2023), hydrothermal vents (Speth et al., 2021; Zhong et al., 2022; Zhou, St. John, et al., 2022), anaerobic digesters and annamox bioreactors (Campanaro et al., 2020; Lin et al., 2021; Nobu et al., 2015; Pabst et al., 2022; Rinke et al., 2013; Suarez et al., 2022; Wang et al., 2020; Wang et al., 2022; Zhang et al., 2022), wastewater (Grégoire et al., 2023; Haryono et al., 2022; Huang et al., 2021; Parks et al., 2017) and saline and hypersaline sediments (Uzun et al., 2020; Vavourakis et al., 2019). Of these studies, only one characterized this organism and identified Ca. Hydrogenedentota as a syntrophic degrader of lipids and glycerol within a terephthalate degrading bioreactor (Nobu et al., 2015). Although this Hydrogenedentota microbe only constituted 0.8% of the community, its transcriptome represented 10% of the total sequenced bacterial transcriptome, supporting a substantive role of Hydrogenedentota in the carbon flux within this system (Nobu et al., 2015).

Microbes belonging to the Candidate phylum Sumerlaeota have previously been identified in Shark Bay microbial mats and microbialites using 16S rRNA amplicon sequencing (Wong et al., 2015) and metagenomic sequencing (Babilonia et al., 2018; Chen et al., 2020; Kindler et al., 2022; Skoog et al., 2022). To the best of our knowledge, only one Sumerlaeota MAG has been assembled from a pustular mat in Shark Bay (Skoog et al., 2022). MAGs of Sumerlaeota have been assembled from other environments including other hypersaline microbial mats and marine biofilms (Karačić et al., 2018), anaerobic digesters and anammox bioreactors (Mei, 2020; Suarez et al., 2022; Wang et al., 2020; Ya et al., 2022; Zorz, 2021), animal gut microbiomes (Lemos et al., 2022), freshwater lake water and sediment (Hahn et al., 2022; Jaffe et al., 2023; Tran et al., 2021), coral skeleton biofilms (Tandon et al., 2022), hot spring sediment and water (Fang et al., 2021; Kato et al., 2022; Liew et al., 2022), water and sediments around a hydrothermal vent (Speth et al., 2021; Zhong et al., 2022), marine, saline, and hypersaline sediments (Baker et al., 2015; Fang et al., 2021; Rinke et al., 2013; Vavourakis et al., 2019; Zhou, Mara, et al., 2022), subsurface water (He et al., 2020; Hernsdorf et al., 2017; Kadnikov et al., 2019), soil (Parks et al., 2018), wastewater (Haryono et al., 2022; Huang et al., 2021), and landfills (Grégoire et al., 2023). This rare candidate phylum appears to be omnipresent but is always reported at very low abundances (<1%). Until the present work, only two studies have characterized MAGs of these organisms: one from a deep subsurface aquifer (Kadnikov et al., 2019) and another from geothermal springs and saline lake sediments (Fang et al., 2021). Both studies have characterized Ca. Sumerlaeota microbes as facultative anaerobes that metabolize chitin among other carbohydrate substrates (Fang et al., 2021; Kadnikov et al., 2019).

Here, we assess the roles of Hydrogenedentota and Sumerlaeota in a pustular mat from Shark Bay, WA, by characterizing the metabolic potential of these two organisms within this system. We compare these metabolic findings to assessed core metabolic capabilities of Hydrogenedentota and Sumerlaeota MAGs from other environments, determine the potential adaptations of Sumerlaeota and Hydrogenedentota MAGs to osmotic stress, heavy metal toxicity, nutrient limitation, oxidative stress and ultraviolet (UV) radiation in Shark Bay, and identify adaptations of the Sumerlaeota and Hydrogenedentota microbes to these environmental stresses across environments.

## RESULTS AND DISCUSSION

Whole genomic DNA was extracted from a pustular mat collected from Carbla Beach, Shark Bay, WA, sequenced using an Illumina NextSeq sequencer, and analysed according to Skoog et al. (2022). Briefly, DNA sequence fragments were quality filtered and trimmed using Trimmomatic v.0.36 (Bolger et al., 2014), assembled with MEGAHIT v1.0.2. (Li et al., 2015; Li et al., 2016), binned using MetaBAT v2.12.1 (Kang et al., 2015), and quality filtered using CheckM (Parks et al., 2015). This analysis yielded 83 MAGs that were taxonomically classified using GTDB‐Tk v0.2.2 (Chaumeil et al., 2020). One of these MAGs was assigned to the candidate phylum Hydrogenedentota (MAG 71) and another MAG was classified as the candidate phylum Sumerlaeota (MAG 30; [Skoog et al., 2022]). CheckM results revealed that MAG 71 is 72.03% complete and 0% contaminated and identified MAG 30 as 96.07% complete and 2.45% contaminated. CoverM (v0.6.1) was used to determine relative abundances of 0.13% and 0.23% of Hydrogenedentota (MAG 71) and Sumerlaeota (MAG 30) within the pustular mat, respectively, confirming these organisms as members of the rare biosphere in this system (https://github.com/wwood/CoverM).

### Putative carbon metabolisms

**FIGURE 1:**
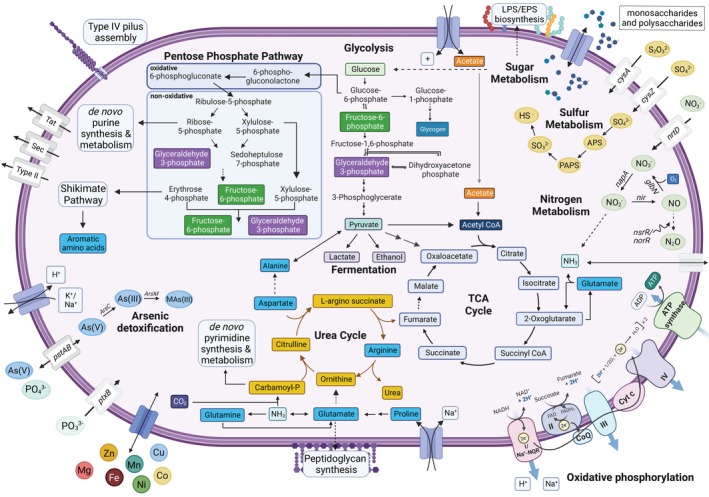
Metabolic reconstruction of Ca. Sumerlaeota MAG 30. MAG 30 was annotated with Prokka (v1.14.6) using default parameters (Seemann, 2014) as well as using the Department of Energy (DOE) Joint Genome Institute Integrated Microbial Genomes (JGI IMG) Annotation Pipeline v.4.16.5 (Huntemann et al., 2015; Markowitz et al., 2007). Solid arrows indicate gene presence; dashed arrows indicate the absence of a gene within a pathway.

**FIGURE 2:**
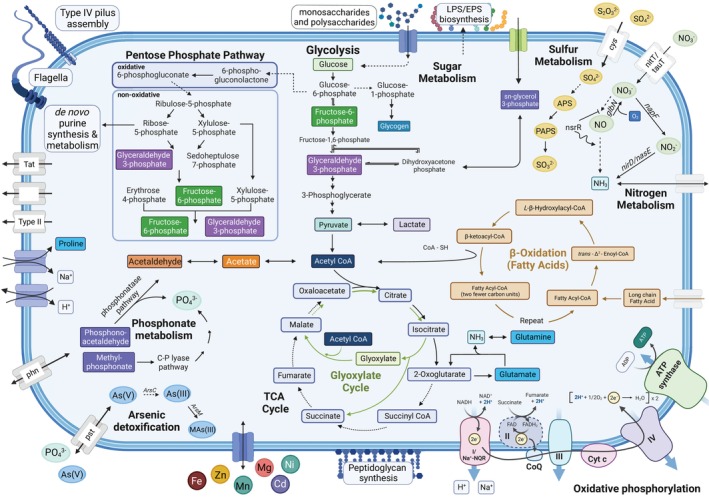
Metabolic reconstruction of Ca. Hydrogenedentota MAG 71. MAG 71 was annotated with Prokka (v1.14.6) using default parameters (Seemann, 2014) as well as using the Department of Energy (DOE) Joint Genome Institute Integrated Microbial Genomes (JGI IMG) Annotation Pipeline v.4.16.5. (Huntemann et al., 2015; Markowitz et al., 2007). Solid arrows indicate gene presence; dashed arrows indicate the absence of a gene within a pathway.

Metabolic reconstruction of Sumerlaeota MAG 30 and Hydrogenedetota MAG 71 suggests facultatively anaerobic, chemoorganotrophic lifestyles for these organisms. MAG 30 possessed a complete Embden‐Meyerhof‐Parnas (EMP) glycolysis pathway, a near‐complete TCA cycle and oxidative and non‐oxidative pentose phosphate pathway (PPP) and all genes necessary for oxidative phosphorylation, suggesting the capability for aerobic respiration. This Sumerlaeota representative also possessed lactate dehydrogenase (LDH) and alcohol dehydrogenase (ADH) enzymes, suggesting the potential for facultative anaerobiosis (Figure 1; Table S1). The incompleteness (72%) of MAG 71 may explain the presence of only near‐complete glycolysis, oxidative and non‐oxidative PPP, and oxidative phosphorylation pathways (Figure 2; Table S1). The genome of the Hydrogenedentota representative did not encode for a complete TCA cycle but did contain all five genes involved in the glyoxylate cycle. The use of the glyoxylate shunt as an alternative to the TCA cycle may help MAG 71 circumvent iron limitation and respond to oxidative and antibiotic stress (Ahn et al., 2016; Koedooder et al., 2018) in Shark Bay. The presence of LDH in this MAG also suggests its ability to respire anaerobically (Table S1). The ability of both MAGs to adapt to varying oxygen concentrations may enable these organisms to occupy the spatiotemporally variable redox niches in pustular mats from Shark Bay (Pages et al., 2014; Revsbech et al., 1983).

A nearly complete fatty acid ß‐oxidation pathway in the genome of MAG 71 demonstrates the potential of this organisms to metabolize long chain fatty acids. Genes involved in glycerol import and degradation and the interconversion of dihydroxyacetone phosphate and sn‐glycerol 3‐phosphate link lipid metabolism with glycolysis in MAG 71 (Figure 2). The potential of MAG 71 to metabolize long‐chain fatty acids and glycerol is consistent with the noted potential of Hydrogenedentota in wastewater reactors to degrade detrital biomass using this metabolism (Nobu et al., 2015).

Because simple and complex carbohydrates can make up to 90% of the organic carbon in microbial mats (Flemming & Wingender, 2010), the production, modification, and degradation of mono‐, di‐ and polysaccharides can play a major role in carbon cycling within pustular mats (e.g., Skoog et al., 2022). MAG 71 possessed multiple genes involved in the import of ribose, fructose, melibiose and maltose while MAG 30 encoded for the binding and import of lactose, arabinose, maltose and maltodextrin, suggesting the ability of these organisms to import and metabolize these carbohydrates (Table S1). Both MAGs also encoded for genes involved in the modification and degradation of other carbohydrates including starch, rhamnose, mannose, and sucrose as well as genes involved in modifying and metabolizing pullulan, galactose, trehalose, and xylose (MAG 30) and arabinose, fucose, maltose, alginate, and trehalose (MAG 71; Table S1).

The genome of MAG 71 further encoded for ulvan‐active sulfatase, arylsulfatase, chondroitin disaccharide hydrolase, and heparinase genes involved in the specific modification and degradation of sulfated polysaccharides, compounds that are abundant in pustular mats from Shark Bay (Skoog et al., 2022). The presence of arylsulfotransferase and sulfotransferase genes in both MAGs also supported the ability of microbes represented by these MAGs to produce these sulfated polysaccharides in the extracellular matrix of the pustular mats (Skoog et al., 2022). While the degradation of these sulfated polysaccharides can provide a source of carbon, the production of these compounds by these microbes can also confer protection against a number of stresses that microbes face in pustular mat communities (Baba et al., 1988; De Souza et al., 2007; Ghosh et al., 2009; González‐Hourcade et al., 2020; Jayawardena et al., 2020).

MAG 30 also possessed nearly all genes involved in chitin degradation including those involved in hydrolyzing the (1,4)‐*β*‐glycoside bonds of chitin into smaller dimers, those with predicted *β*‐N‐acetyl‐hexosaminidase activity (GH20) which further break these carbohydrates into N‐acetyl‐D‐glucosamine (GlcNAc), and those involved in phosphorylating GlcNAc into GlcNAc‐6‐P for eventual conversion to fructose‐6‐phosphate and incorporation into the EMP glycolytic pathway (Table S1). Diacetylchitobiose uptake genes in both MAGs and an additional chitooligosaccharide deacetylase (*chbG*) in MAG 30 supported potential metabolism of acetylated chitooligosaccharides. A gene encoding for glycosyl hydrolase (GH) family 20 in the genome of MAG 71 also indicated the potential for the degradation of chitooligosaccharides. Both MAG 71 and MAG 30 possessed GH10 genes that display some endo‐*β*‐1,3‐xylanase and endo‐*β*‐1,4‐xylanase activity on aryl cellobiosides (http://www.cazy.org/GH10.html). A gene encoding for cellobiose 2‐epimerase in MAG 30 further supports the utilization of this substrate. MAG 30 possessed additional genes (endoglucanase, cellulase and GH5) involved in the degradation of cellulose into short‐chain cello‐oligosaccharides as well as *β*‐glucosidases involved in breaking down cello‐oligosaccharides into glucose and glucose‐6‐phosphate, a substrate for glycolysis. The ability of MAG 30 to degrade chitin and cellulose aligns with previous descriptions of this candidate phylum (Fang et al., 2021; Kadnikov et al., 2019), but the potential of Hydrogenedentota MAG 71 to degrade cellulose and chitin derivatives was not recognized previously. In Shark Bay, chitin may be derived from some diatoms, chlorophyta, cyst walls of some flagellated, ciliated and amoeboid protozoans, fungal cell walls or invertebrate exoskeletons (Durkin et al., 2009; Field et al., 1998; Mulisch, 1993; Nelson et al., 1995), whereas cellulose is likely of algal or terrestrial origin (Bogolitsyn et al., 2020; Han et al., 2022; Walker & McComb, 1985).

### Inferred nitrogen metabolisms

Nitrogen is essential for growth of all organisms and can also be used in dissimilatory metabolism. Consequently, both MAG 71 and MAG 30 encode for numerous pathways for the transport and metabolism of nitrogen compounds. MAG 71 contained genes encoding for nitrate transport and nitrite import and assimilation. MAG 71 possessed the ability to interconvert glutamate and glutamine and excrete ammonia by‐products through ammonium transporters (Figure 2; Table S1). Genes involved in nitrate import and reduction were present in MAG 30 (Table S1). Recent studies have revealed the role of *napA* in dissimilatory nitrate reduction under anaerobic conditions indicating that MAG 30 may perform dissimilatory reduction of nitrate to nitrite (Alcántara‐Hernández et al., 2009; Chen et al., 2022). Moreover, the presence of *nir* genes indicates the denitrifying capabilities of this Sumerlaeota MAG. Genes involved in the breakdown and biosynthesis of glutamate were also present in both MAGs as were the carbamoyl‐phosphate synthase genes that convert ammonia and bicarbonate to carbamoyl phosphate. Previous metabolic analyses of microbial mat communities in Shark Bay suggest an incomplete nitrogen cycle resulting in the build‐up of potentially toxic ammonia (Ruvindy et al., 2016). Carbamoyl phosphate produced from ammonia can be fed to the urea cycle encoded in MAG 30 as a potential means for ammonia detoxification (Figure 1). MAG 30 also possessed the capability to denitrify and metabolize various nitrogen compounds including nitronates and nitriles (Table S1). Taxa and metabolic pathways associated with the biogeochemical cycling of nitrogen in Shark Bay microbial mats point to Bacteroidetes, Alpha‐ and Deltaproteobacteria as primarily responsible for denitrification (Ruvindy et al., 2016; Wong et al., 2018). The present study sheds light on Sumerlaeota members of the rare biosphere as additional players in the dissimilatory reduction of nitrate within these communities.

Nitric oxide (NO) is a key signalling molecule within living organisms, but is also a highly reactive nitrogen intermediate within the denitrification pathway (Toyofuku & Yoon, 2018; Wilbert & Newman, 2022). Both genomes possessed transcriptional repressors (*nsrR*) involved in sensing nitric oxide (Tucker et al., 2008). MAG 30 also contained nitrogen regulatory and response proteins (*ntrC*, *glnG*) and an anaerobic nitric oxide reductase transcription regulator (*norR*), all linked to bacterial nitric oxide detoxification and response to nitrosative stress (Figure 1; [D'Autréaux et al., 2005; Gardner et al., 2003; Leigh & Dodsworth, 2007; Mukhopadhyay et al., 2004]). Verrucomicrobia, Proteobacteria, Planctomycetes, and Myxococcota MAGs within this pustular mat community also encoded for *norR*, but no other phyla possessed genes encoding for *nsrR* or *ntrC*. Both MAGs also contained Group 1 truncated hemoglobins (*glbNs)* with a demonstrated role in intracellular NO detoxification by oxidizing NO to nitrate (Figures 1 and 2; [Milani et al., 2003; Ouellet et al., 2002; Sanz‐Luque et al., 2015]). *glbNs* were also identified in Verrucomicrobia and Planctomycetes MAGs from the community, suggesting the potential exposure of all these microorganisms to NO. The organism represented by MAG 30 may use *glbN* to remove NO created through an encoded denitrification cycle while MAG 71 may use this mechanism to detoxify NO that diffuses into cells or is produced by metabolic processes that are not evident in this incomplete genome. MAG 30 possesses the capability to cycle nitrogen through denitrification and metabolize various nitrogen compounds including nitronates and nitriles – a beneficial adaption to potential nitrate‐limiting conditions – and potentially detoxify ammonia by producing urea (Figure 1; Table S1).

### Inferred sulfur metabolisms

Sulfate reduction is a measurable and prominent metabolism in the various microbial mats in Shark Bay (Campbell et al., 2020; Pages et al., 2014; Wong et al., 2018). Neither MAG 30 nor MAG 71 is capable of dissimilatory sulfate reduction, but these MAGs, respectively, encoded for complete and near‐complete pathways for assimilatory sulfate reduction (Figures 1 and 2). The presence of several sulfatase and arylsulfatase genes in MAG 71 also suggested that this organism could scavenge organic sulfur from sulfated polysaccharides that are present within the Shark Bay pustular mat (Table S1; [Skoog et al., 2022]).

### Inferred phosphorous metabolisms

Phosphorus is essential for cell growth and production of phospholipids, proteins and polysaccharides. Microbes assimilate phosphorus primarily as inorganic phosphate (PO_4_^3−^); however, phosphate is limiting (~3–6 μM) in Shark Bay (Atkinson, 1987; Pages et al., 2014; Wong et al., 2018). Metabolic reconstruction identified MAG 71 and MAG 30 as well‐adapted to these phosphate‐limited conditions. Both MAGs encoded for phosphate transporters (*pstAB‐phoU, phoT*) and phosphate starvation‐inducible proteins (*phoH*). MAG 30 also possessed genes encoding for the most common bacterial version of the two‐component regulatory system (phoR–phoB) of the inducible phosphate starvation sensor regulon (*pho, pst*; [Santos‐Beneit, 2015]). MAG 71 contained *ppk* involved in the breakdown of polyphosphate (polyP) and encoded for both main phosphonate utilization mechanisms—hydrolysis by phosphonatase (*pnhXZ*) and C‐P lyase pathways (*phnHIJLM*)—suggesting the potential importance of phosphonate metabolism in the ability of Hydrogenedentota to assimilate phosphorus in the nutrient‐limited environment of Shark Bay (Kononova & Nesmeyanova, 2002). Assimilation of inorganic polyPs have also been associated with microbial resistance to high salinity, heavy metal toxicity and oxidative stress, which may be beneficial for this organism in combating multiple stresses in Shark Bay (Ayraud et al., 2005; Kornberg et al., 1999; Ogawa et al., 2000; Sureka et al., 2007).

### Adaptations to environmental stresses in Shark Bay

Pustular microbial mats in Shark Bay, WA, are exposed to multiple environmental stressors including oxidative stress and UV radiation (Ruvindy et al., 2016; Wong et al., 2015; Wong et al., 2018). Multiple genes involved in oxidative stress response in both MAGs demonstrated the ability of these organisms to combat cellular damage caused by reactive oxygen species, free radicals, peroxides, and lipid peroxides produced as a by‐product of microbial photosynthesis and aerobic metabolisms as well as exogenous sources (e.g., xenobiotics and UV radiation; Table S1; [Latifi et al., 2009; Revsbech et al., 1983]). Photolyases encoded in MAG 30 also suggested the ability to repair DNA lesions caused by UV exposure—a stress that pustular mats face regularly (Table S1). MAG 30 encoded for a complete shikimate pathway that supports the production of mycosporine‐like amino acids and other pigments and metabolites that mediate microbial tolerance to UV radiation (Karki & Ham, 2014; Shick et al., 1999). These pathways and genes related to UV response indicate that Sumerlaeota organisms may reside in the photic zone of pustular mats.

Pustular mats in Shark Bay are also exposed to hypersaline waters (Ruvindy et al., 2016; Wong et al., 2015; Wong et al., 2018). Not surprisingly, both MAG 71 and MAG 30 from these mats harbour genes involved in the responses to osmotic stress including genes for the transport and biosynthesis of compatible solutes including glycine betaine, proline and trehalose (Table S1, Figures 1 and 2). Both organisms also possessed Na^+^/proline symporters for passive intake of osmoprotectants and Na^+^/H^+^ antiporters that can regulate ion concentrations within the cell, enabling these organisms to resist cellular damage caused by osmotic stress (Table S1, Figures 1 and 2).

Pustular microbial mats in Shark Bay, WA are also exposed to heavy metals such as arsenic (5.0 μg/L), copper (379.7 μg/L), vanadium (5.0 μg/L), and manganese (3.7 μg/L; [Ruvindy et al., 2016; Wong et al., 2015; Wong et al., 2018]). Genes associated with the cycling of these heavy metals have been previously identified within pustular mat metagenomes from Shark Bay (Babilonia et al., 2018; Ruvindy et al., 2016). We specifically identify genes involved in the transport of various heavy metals including zinc, lead, cobalt, cadmium, manganese, magnesium, nickel, copper and iron in both MAG 71 and MAG 30 (Table S1). Genes regulating the transport of zinc, lead, and manganese under high concentrations were present within MAG 71, suggesting the specific sensitivity of this organism to these metals (Table S1). The presence of high affinity siderophores enterobactin and petrobactin in MAG 30 and MAG 71, respectively, point to the strong ability of these organisms to sequester iron under iron‐limiting conditions (Table S1). Additionally, the presence of arsenate reductase (*arsC*) in MAG 30 and *arsR* and arsenite methyltransferase (*arsM*) in both MAGs identifies the potential for arsenic detoxification through biomethylation of As(III) to methylarsenite (MAs(III); Figures 1 and 2).

### Metabolisms across environments

To understand the metabolic and physiological diversity of the two candidate phyla, we collected all publicly available Hydrogenedentota and Sumerlaeota genomes and MAGs, taxonomically re‐classified and quality‐assessed them, and used them to construct phylogenomic trees supplemented with details on the environmental origins of each organism (Table S2; Figures S1 and S2). Phylogenomic analyses of all publicly available Hydrogenedentota and Sumerlaeota genomes and MAGs revealed some organisms to be grouped by environmental locality, but without any strong trends (Figures S1 and S2). We then compared each of the two MAGs from the pustular microbial mat described in this study to nine other high‐quality and highly‐complete MAGs from the same candidate phyla identified from different environments (Table S2). Although the selection of only one MAG representative of each candidate phylum from different localities does not fully depict the average metabolic capabilities of these microbes, comparisons of these largely complete MAGs provide insights into the metabolic and physiological potentials of these rare biosphere phyla across environments.

**FIGURE 3:**
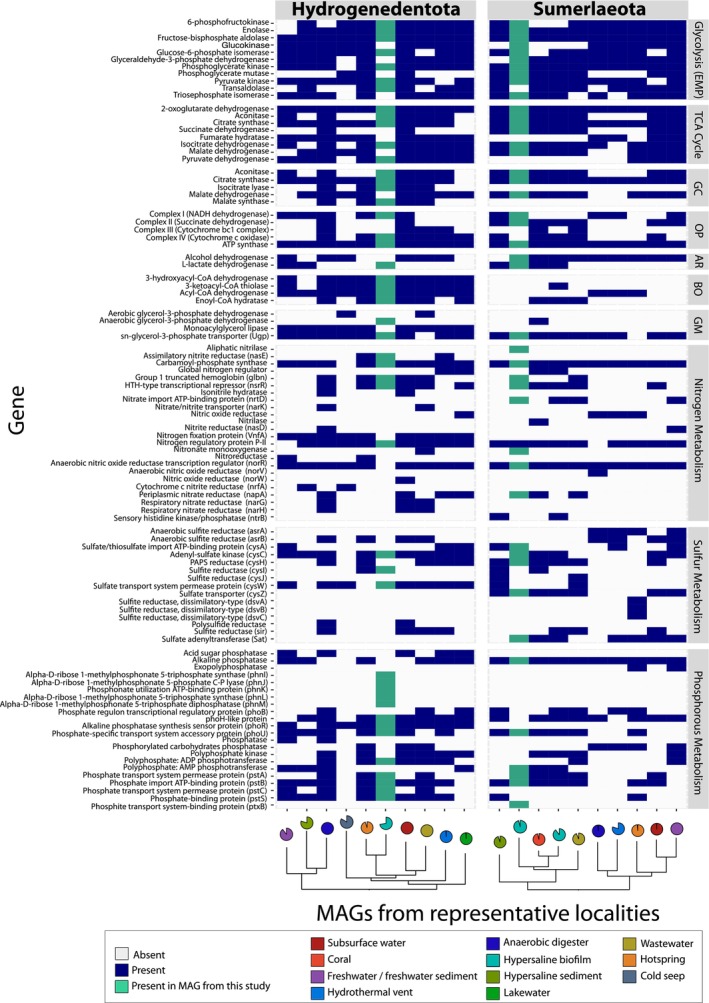
Presence/absence of genes involved in core metabolic pathways of Ca. Sumerlaeota and Ca. Hydrogenedentota environmental representatives. Core metabolisms across sequenced Sumerlaeota and Hydrogenedentota from different environments were compared by selecting one highly complete (>90%, or highest completeness for each representative [Table S2]) MAG from each of the different localities. These select MAGs were subsequently annotated with Prokka (v1.14.6; [Seemann, 2014]). Genes related to core carbon, nitrogen, sulfur and phosphorous metabolisms were identified and visualized using ggplot2 in R (v.3.6.0). Completeness of circles indicate completeness of MAGs. Circle colour indicates environments from which MAGs were obtained. Dendrograms of the selected representative MAGs were constructed using the same methods employed to generate the phylogenomic trees that incorporated all Ca. Sumerlaeota and Ca. Hydrogenedentota organisms (see Figure S1 methodology). AR, anaerobic respiration; BO, beta‐oxidation; GC, glyoxylate cycle; GM, glycerol metabolism; OP, oxidative phosphorylation.

Very few Sumerlaeota and Hydrogenedentota MAG representatives possessed all genes that would demonstrate the capability for facultative anaerobic respiration. The Hydrogenedentota MAG from the subsurface water environment was the only MAG that contained all genes encoding for the EMP glycolytic pathway, TCA cycle, oxidative phosphorylation, and fermentation (Figure 3). The majority of other MAGs representing both candidate phyla exhibited nearly comprehensive gene sets associated with these pathways (Figure 3). The absence of certain genes may be attributed to the varying degrees of completeness among individual MAG representatives; this uncertainty challenges definitive conclusions about the capability of these organisms for aerobic respiration. However, with the exception of Hydrogenedentota MAG representatives from a hot spring and lake water, all MAGs from both candidate phyla encoded for the fermentation of lactate, alcohol or both, indicating the potential of organisms within these candidate phyla to metabolize anaerobically (Figure 3).

Metabolic reconstruction of Hydrogenedentota representatives from various environments revealed the widespread ability of this candidate phylum to metabolize lipids. A majority of Hydrogenedentota representatives appeared capable of fatty acid catabolism via ß‐oxidation (BO), generating acetyl‐CoA to supply the TCA cycle (Figure 3). Sumerlaeota MAGs did not encode for this metabolism. Cold seep and wastewater MAGs of the candidate phylum Hydrogenedentota possessed genes involved in glycerol import and degradation under aerobic conditions (*glpD*; [Ingledew & Poole, 1984; Iuchi et al., 1990]), whereas Hydrogenedentota MAG 71 possessed the metabolic capability to metabolize glycerol anaerobically (*glpC*), using fumarate as the terminal electron acceptor in the oxidation of glycerol (Ehrmann et al., 1987; Ingledew & Poole, 1984). These findings support previous studies that identified these organisms as degraders of detrital biomass in wastewater reactors (Nobu et al., 2015). These organisms may serve a similar role in other niches as well.

The genomes of organisms from both phyla presented evidence for additional respiratory metabolisms. Sumerlaeota representatives from anaerobic digester, hydrothermal vent, and freshwater environments possessed both subunits of the assimilatory sulfite reductase *asrAB* (Figure 3). The Sumerlaeota hot spring MAG representative encoded for the dissimilatory sulfite reductase *dsrABC*, revealing the potential for this organism to perform dissimilatory sulfate reduction. No MAG representatives of the Hydrogenedentota phyla possessed these genes. The dissimilatory nitrate reductase, *napA*, was present in Hydrogenedentota genomes from anaerobic digester, subsurface water, hydrothermal vent and lakewater environments and in Sumerlaeota MAGs from wastewater, Shark Bay and coral skeleton localities (Figure 3). Thus, organisms from these phyla may respire nitrate across diverse environments. Representatives of Hydrogenedentota from anaerobic digester, subsurface water, and wastewater also possessed *narGH* genes, further suggesting the ability of some Hydrogenedentota members to respire nitrate. Other Hydrogenedentota MAG representatives from hypersaline sediments and cold seep environments possess a lesser studied dissimilatory nitrite reductase (*nrfA*), predicted to play a role in specifically nitrate‐ and nitrite‐limited environments (Wang & Gunsalus, 2000).

Genes involved in the assimilation of sulfite, nitrite and nitrate provide additional clues that microbes from both phyla can metabolize under anaerobic conditions. Hydrogenedentota MAG representatives from subsurface water and anaerobic digester, but not from Shark Bay, possessed the capacity to perform assimilatory sulfite reduction (SIR). Some Hydrogenedentota and Sumerlaeota MAG representatives also encoded for assimilatory nitrite reductases, *nasE* and *nasD* (Figure 3). Nitrogen regulatory proteins in all analysed MAGs with the exception of the freshwater Hydrogenedentota MAG and the Sumerlaeota MAG from an anaerobic digester underscored the importance of nitrogen acquisition and metabolic regulation in these phyla (Figure 3).

### Adaptations to stress across environments

**FIGURE 4:**
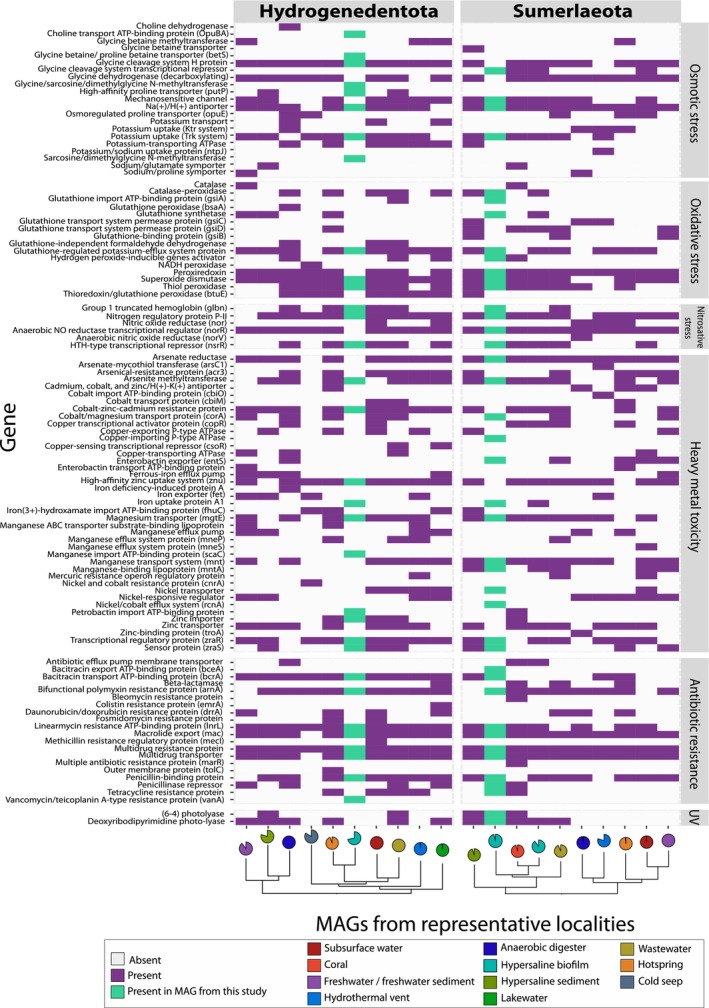
Genes involved in stress adaptation and responses of Ca. Sumerlaeota and Ca. Hydrogenedentota environmental representatives. Stress metabolisms across sequenced Sumerlaeota and Hydrogenedentota from different environments were compared by selecting one highly complete (>90%, or highest completeness for each representative [Table S2]) MAG from each of the different localities. These select MAGs were subsequently annotated with Prokka (v1.14.6; [Seemann, 2014]). Genes related to stress metabolisms were identified and visualized using ggplot2 in R (v.3.6.0). Completeness of circles indicate completeness of MAG. Circle colour indicates environments from which MAGs were obtained. Dendrograms of the selected representative MAGs were constructed using the same methods employed to generate the phylogenomic trees that incorporated all Ca. Sumerlaeota and Ca. Hydrogenedentota organisms (see Figure S1 methodology).

The abundance of genes involved in NO metabolism in multiple Sumerlaeota and Hydrogenedentota MAG representatives suggested the exposure and adaptation of these organisms to nitrosative stress across several environments in addition to Shark Bay. Several genomes including Sumerlaeota from Shark Bay and wastewater environments, and Hydrogenedentota from an anaerobic digester, hot spring, Shark Bay, wastewater and subsurface water contained *glbNs*, emphasizing the potential exposure of these organisms to NO and the importance for these communities to detoxify intracellular NO by oxidation to nitrite (Figure 4). *NorV* and the co‐transcribed *norW* gene are involved in NO detoxification under anaerobic and microaerobic growth conditions (Gardner et al., 2002; Gardner et al., 2003; Hutchings et al., 2002) and we noted the presence of *norV* in the Sumerlaeota genome representative from an anaerobic digester and *norW* within the Hydrogenedentota representative from subsurface water (Figure 4). All MAG representatives, with the exception of MAG 71 from Shark Bay, encoded for the anaerobic nitric oxide reductase transcription regulator, *norR*, further highlighting the importance of NO regulation and detoxification for members of both candidate phyla.

All analysed representative MAGs contained genes involved in combating several environmental stresses (Figure 4). Analyses reveal that all Sumerlaeota and Hydrogenedentota MAG representatives, including those from freshwater environments, possessed a combination of genes involved in synthesizing or transporting osmoprotectants including glycine betaine, proline, trehalose and proline betaine (Figure 4). The influx and accumulation of sodium, potassium and compatible solutes as strategies for combating osmotic stress have previously been reported in Sumerlaeota representatives (Fang et al., 2021), but not in Hydrogenedentota. Multiple mechanisms by which Sumerlaeota and Hydrogenedentota can combat osmotic stress revealed potential adaptations of these organisms to saline and hypersaline environments. Nearly all genomes also possessed peroxidases, glutathione transporters and superoxide dismutase. About half of the genomes from each phylum also contained genes involved in protection against UV stress and were not restricted to environment type (Figure 4).

All analysed representative MAGs contained a variety of genes related to the transport of heavy metals including zinc, lead, copper, nickel, cobalt, mercury, iron, cadmium and arsenic (Figure 4). With the exception of Hydrogenedentota MAGs sequenced from Shark Bay and the cold seep environment, all MAGs representatives of both candidate phyla possessed arsenate reductases, suggesting the need for arsenic detoxification (Figure 4). All Hydrogenedentota MAGs, with the exception of the cold seep and freshwater Hydrogenedentota genomes, also possessed an arsenite methyltransferase gene, further emphasizing the importance for arsenic detoxification in these phyla (Figure 4). Hydrogenedentota MAGs from hypersaline sediment, subsurface water, Shark Bay and wastewater as well as Sumerlaeota MAGs from hypersaline sediment, Shark Bay and a hot spring possessed both genes involved in the ZraSR two‐component system implicated in resistance to lead and zinc under high extracellular zinc and lead concentrations. The Hydrogenedentota genome from an anaerobic digester had a specific gene for the tolerance of mercury that was not found in Hydrogenedentota MAGs from other environments. Additionally, genes involved in the transport of the siderophores petrobactin and enterobactin in Hydrogenedentota MAGs from a bioreactor, freshwater, and Shark Bay and Sumerlaeota MAGs from Shark Bay, coral, wastewater, subsurface water, freshwater and a hot spring identified the adaptations of these organisms to iron limitation in various surface, subsurface and industrial systems (Figure 4).

Nearly all Sumerlaeota and Hydrogenedentota MAG representatives also encoded for the transport and regulation of specific antibiotic metabolites (Figure 4). Organisms also possessed a combination of multidrug resistance proteins that can confer resistance against novobiocin, deoxycholate, norfloxacin, ciprofloxacin, ethidium, kanamycin, polymyxin, linearmycin, bacitracin, streptomycin and β‐lactam antibiotics (Table S1). These antibiotic resistance genes play an especially crucial role for organisms thriving in biofilm‐associated environments where microorganisms are consistently exposed to antimicrobial agents produced by neighbouring community members (Mah, 2012). These genomes also possessed multidrug and AcrAB‐TolC efflux systems capable of pumping out various types of antibiotics such as vancomycin, penicillin and general macrolides (Table S1). These AcrAB‐TolC efflux systems have also been shown to play an important role in biofilm formation, further supporting the potential biofilm‐associated lifestyles of these candidate phyla (Alav et al., 2018; Baugh et al., 2014; Bay et al., 2017).

### Life in biofilm communities

Identifications of Hydrogenedentota and Sumerlaeota across a multitude of biofilm‐associated environments (e.g., microbial mats, bioreactors, hydrothermal vents, cold seeps, hot springs, sediments, marine biofilms, coral, etc.) suggest a potential association of these candidate phyla with biofilm habitats. The metabolic capabilities and stress response findings of this study also suggest that these candidate phyla may be particularly adapted to life in biofilm communities. Biofilm matrices are typically composed of extracellular polymeric substances (EPS) comprised of simple to complex carbohydrates (Flemming & Wingender, 2010). The genomic capacities for both candidate phyla to metabolize varying polysaccharides suggests the potential roles of these organisms in EPS cycling within biofilm‐associated communities. The ability of both organisms to produce sulfated polysaccharides and Hydrogenedentota to degrade these compounds may highlight the specific role of both candidate phyla in the cycling of sulfated polysaccharides that have been implicated as important in shaping and protecting the mat communities in Shark Bay (Skoog et al., 2022). Evidence for the potential of Hydrogenedentota to additionally degrade glycerol and fatty acids across multiple biomes indicates that these organisms may contribute to the cycling of carbon in biofilms by growing on lysed microbial cells and degrading the EPS matrix.

Biofilm communities can harbour synergistic consortia, while simultaneously demanding the ability of each organism to tolerate the antimicrobial agents and by‐products produced by adjacent community members. Therefore, the identification of multiple antibiotic resistance genes in these candidate phyla is consistent with the adaptation of these organisms to living in biofilm‐associated environments (Mah, 2012). The ability of these candidate phyla to sense and detoxify NO may also be an adaptational strategy for coping with NO produced by neighbouring community members, further highlighting the adaptations of these organisms to biofilm communities.

## CONCLUSION

This work characterizes the metabolic and physiological potentials of Sumerlaeota and Hydrogenedentota MAGs from a microbial mat from Shark Bay and compares and contrasts them to MAGs of the same phyla from a range of microbial systems. Metabolic reconstruction and comparisons across environments suggest that both candidate phyla can degrade carbohydrates and respire under varying oxygen concentrations. Ca. Hydrogenedentota can degrade phosphonates and fatty acids and may contribute to the metabolism of lysed microbial biomass in pustular mats and other niches. Evidence for denitrification within the Sumerlaeota candidate phylum also identifies the potential role of these organisms in nitrogen cycling within their environments. Both candidate phyla are well adapted to polyextreme environments and possess mechanisms for coping with osmotic stress, oxidative stress, heavy metal toxicity, nitrosative stress, and antibiotic exposure. Adaptations to these environmental stresses and the capability to degrade carbohydrates, which are common in the matrix of diverse biofilms, may explain why Hydrogenedentota and Sumerlaeota are both commonly detected, while still rare, in biofilms across various environments.

## AUTHOR CONTRIBUTIONS

**Emilie J. Skoog:** Conceptualization (lead); data curation (lead); formal analysis (lead); investigation (lead); methodology (lead); visualization (lead); writing – original draft (lead); writing – review and editing (equal). **Tanja Bosak:** Funding acquisition (lead); project administration (equal); supervision (lead); writing – review and editing (equal).

## CONFLICT OF INTEREST STATEMENT

The authors declare no conflicts of interest.

## Supporting information


**Figure S1.** Phylogenomic tree of Ca. Hydrogenedentota. All 104 publicly available MAGs and genomes previously identified as Ca. Hydrogenedentota were taxonomically re‐classified with GTDB‐Tk (v2.1.1; [Chaumeil et al., 2020]) and assessed for completeness and contamination using CheckM (v1.2.2; [Parks et al., 2015]; Table S2). All genomes with completeness scores <50% and >10% contamination were removed from further analyses and are not included in the phylogenomic tree (see Table S2). Anvi'o (v7.1) was used to generate a contig database for each MAG and identify sequences based on the hmm search for all bacterial genes within the Bacteria_71 bacterial single‐copy core gene collection (Eren et al., 2015). This generated a concatenated protein file then used to construct maximum‐likelihood phylogenetic trees which were generated using IQTree (v1.6.3) run with ModelFinder Plus (MFP) testing the following base models: LG+, WAG+ and BLOSUM62 [Nguyen et al., 2015]. Support for bipartitions was determined using rapid bootstraps (1000 replicates) and SH‐aLRT tests (1000 replicates). Trees were mid‐point rooted, and FigTree (v.1.4.3) was used to visualize the resulting trees. Node colours indicate bootstrap value ranges according to the legend. Red star denotes the Hydrogenedentota (MAG 71) representative from Shark Bay. Branch colours indicate environments from which different organisms were sequenced. Updated taxonomic classification of all publicly available Hydrogenedentota MAGs assigned several MAGs to different candidate phyla which are seen grouped outside of the Hydrogenedentota group (see Table S2). Taxonomic classifications are presented at the most fundamental levels of taxonomy (see Table S2).

**Figure S2.** Phylogenomic tree of Ca. Sumerlaeota. All 60 publicly available MAGs and genomes previously identified as Ca. Sumerlaeota were taxonomically re‐classified with GTDB‐Tk (v2.1.1; [Chaumeil et al., 2020]) and assessed for completeness and contamination using CheckM (v1.2.2; [Parks et al., 2015]; Table S2). All genomes with completeness scores <50% and > 10% contamination were removed from further analyses and are not included in the phylogenomic tree (see Table S2). Phylogenomic analysis were performed in the same manner as that of Ca. Hydrogenedentota (see Figure S1 methodology). Red star denotes the Sumerlaeota (MAG 30) representative from Shark Bay. Branch colours indicate environments from which different organisms were sequenced. Node colours indicate bootstrap value ranges according to the legend. Updated taxonomic classification of all publicly available Sumerlaeota MAGs assigned several MAGs to different candidate phyla which are seen grouped outside of the Sumerlaeota group (see Table S2). Taxonomic classifications are presented at the most fundamental levels of taxonomy (see Table S2).

**Table S1.** List of genes in Ca. Hydrogenedentota MAG 71 and Ca. Sumerlaeota MAG 30. MAGs were annotated with Prokka (v1.14.6) using default parameters (Seemann, 2014) as well as using the Department of Energy (DOE) Joint Genome Institute Integrated Microbial Genomes (JGI IMG) Annotation Pipeline v.4.16.5 (Huntemann et al., 2015; Markowitz et al., 2007).

**Table S2.** Identification, taxonomic analysis and quality assessment of all publicly available Ca. Hydrogenedentota and Ca. Sumerlaeota. All publicly available MAGs and genomes previously identified as Ca. Hydrogenedentota and Ca. Sumerlaeota were taxonomically re‐classified with GTDB‐Tk (v2.1.1; [Chaumeil et al., 2020]) and assessed for completeness and contamination using CheckM (v1.2.2; [Parks et al., 2015]). All genomes with completeness scores <50% and >10% contamination were removed from further analyses and are included at the bottom of the table.

## Data Availability

Raw sequence data used to assemble the two MAGs from Shark Bay and assembled MAGs are publicly available on Zenodo (https://zenodo.org/doi/10.5281/zenodo.3874995) and on the Joint Genome Institute website under the GOLD AP ID Ga0316160. All supplementary material and phylogenomic tree files are in figshare: https://doi.org/10.6084/m9.figshare.24438637.v1.
