# Supplementary figures and images for: Predicted metabolic roles and stress responses provide insights into candidate phyla Hydrogenedentota and Sumerlaeota as members of the rare biosphere in biofilms from various environments
Source: Environ Microbiol Rep. 2024 Jan 9;16(1):e13228. doi: 10.1111/1758-2229.13228 (PMC10866078; doi:10.1111/1758-2229.13228)

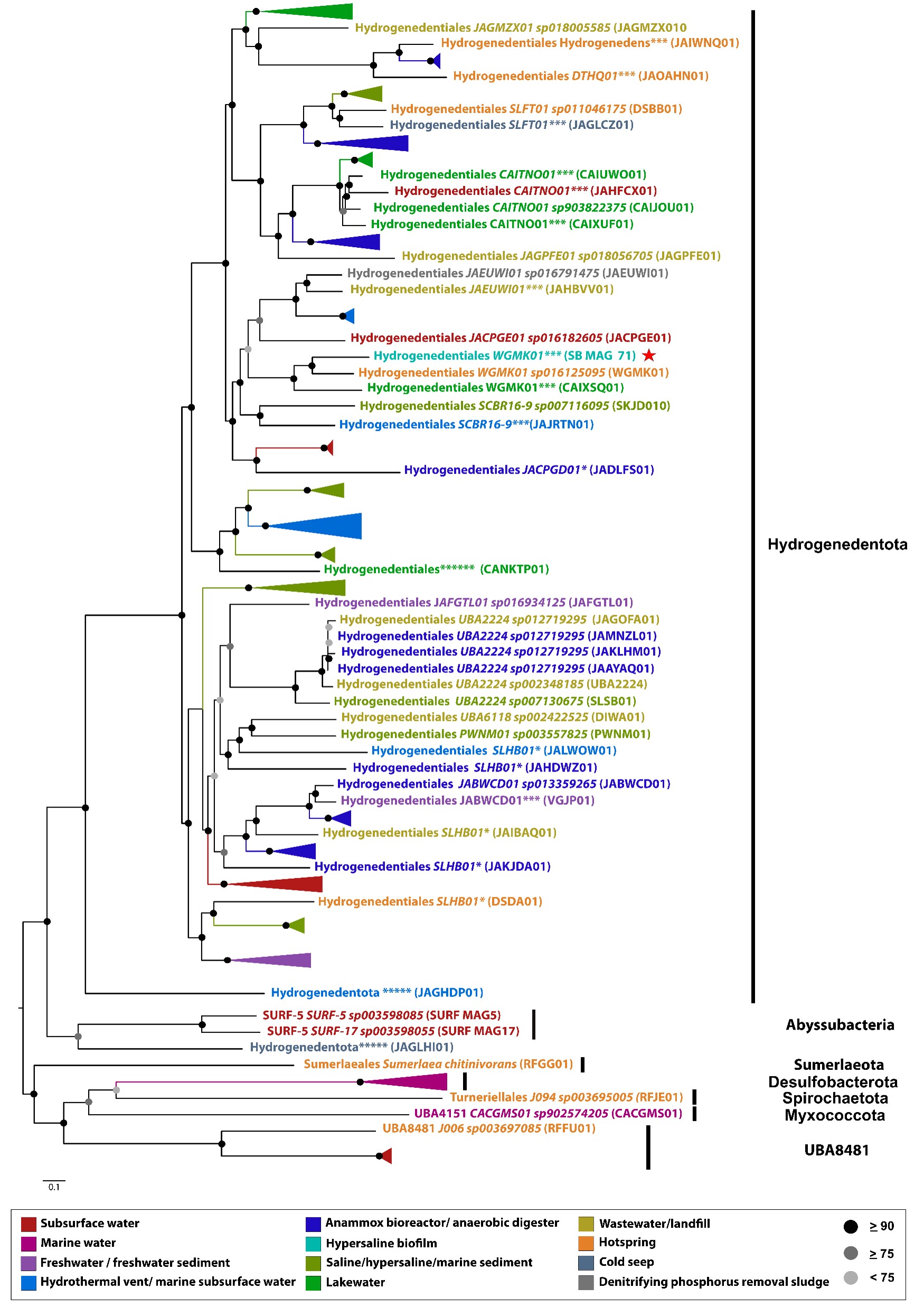

Supplement: Supplementary file 1 — Figure S1. Phylogenomic tree of Ca. Hydrogenedentota. All 104 publicly available MAGs and genomes previously identified as Ca. Hydrogenedentota were taxonomically re‐classified with GTDB‐Tk (v2.1.1; [Chaumeil et al., 2020]) and assessed for completeness and contamination using CheckM (v1.2.2; [Parks et al., 2015]; Table S2). All genomes with completeness scores <50% and >10% contamination were removed from further analyses and are not included in the phylogenomic tree (see Table S2). Anvi'o (v7.1) was used to generate a contig database for each MAG and identify sequences based on the hmm search for all bacterial genes within the Bacteria_71 bacterial single‐copy core gene collection (Eren et al., 2015). This generated a concatenated protein file then used to construct maximum‐likelihood phylogenetic trees which were generated using IQTree (v1.6.3) run with ModelFinder Plus (MFP) testing the following base models: LG+, WAG+ and BLOSUM62 [Nguyen et al., 2015]. Support for bipartitions was determined using rapid bootstraps (1000 replicates) and SH‐aLRT tests (1000 replicates). Trees were mid‐point rooted, and FigTree (v.1.4.3) was used to visualize the resulting trees. Node colours indicate bootstrap value ranges according to the legend. Red star denotes the Hydrogenedentota (MAG 71) representative from Shark Bay. Branch colours indicate environments from which different organisms were sequenced. Updated taxonomic classification of all publicly available Hydrogenedentota MAGs assigned several MAGs to different candidate phyla which are seen grouped outside of the Hydrogenedentota group (see Table S2). Taxonomic classifications are presented at the most fundamental levels of taxonomy (see Table S2). [file EMI4-16-e13228-s002.jpg]

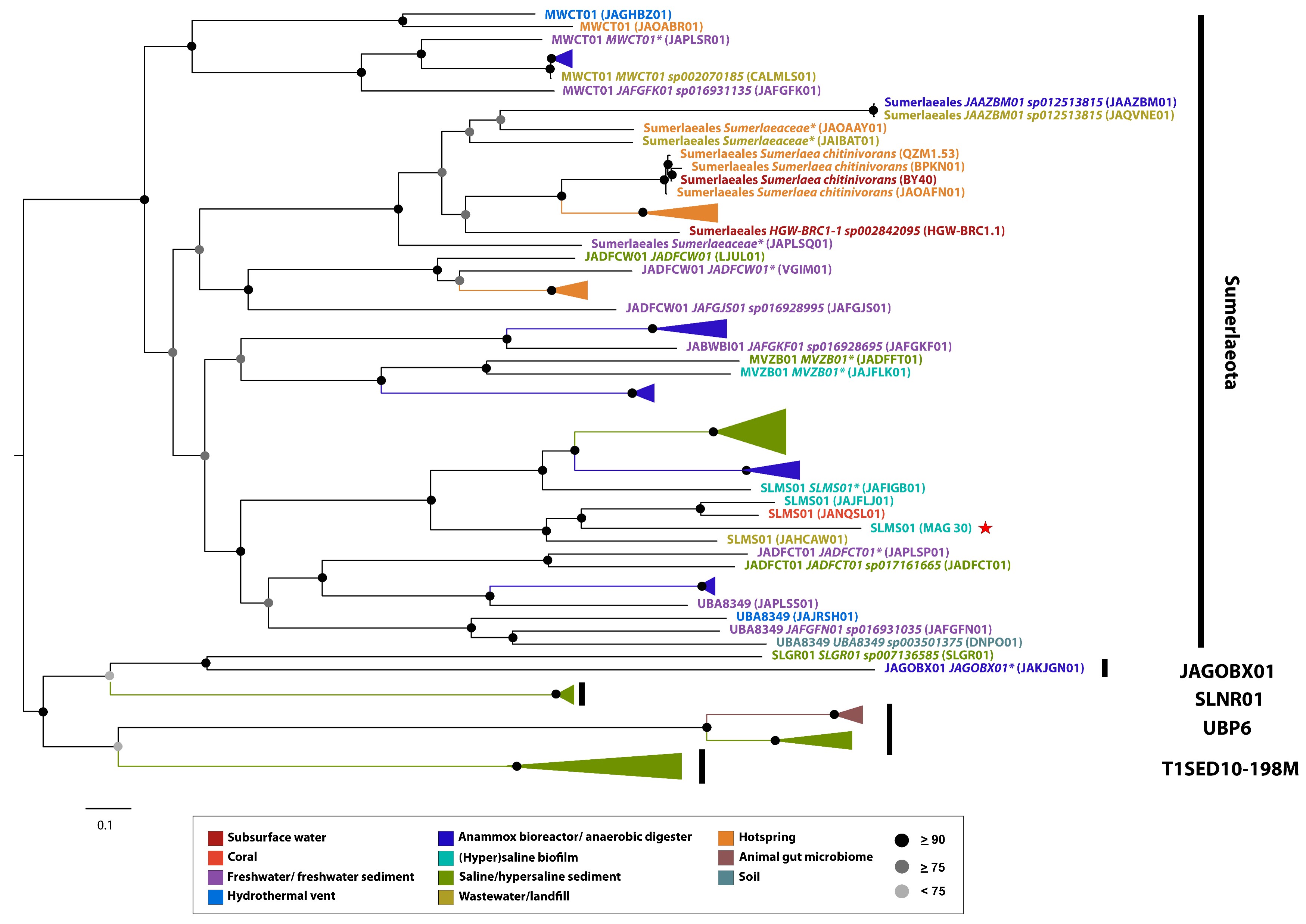

Supplement: Supplementary file 2 — Figure S2. Phylogenomic tree of Ca. Sumerlaeota. All 60 publicly available MAGs and genomes previously identified as Ca. Sumerlaeota were taxonomically re‐classified with GTDB‐Tk (v2.1.1; [Chaumeil et al., 2020]) and assessed for completeness and contamination using CheckM (v1.2.2; [Parks et al., 2015]; Table S2). All genomes with completeness scores <50% and > 10% contamination were removed from further analyses and are not included in the phylogenomic tree (see Table S2). Phylogenomic analysis were performed in the same manner as that of Ca. Hydrogenedentota (see Figure S1 methodology). Red star denotes the Sumerlaeota (MAG 30) representative from Shark Bay. Branch colours indicate environments from which different organisms were sequenced. Node colours indicate bootstrap value ranges according to the legend. Updated taxonomic classification of all publicly available Sumerlaeota MAGs assigned several MAGs to different candidate phyla which are seen grouped outside of the Sumerlaeota group (see Table S2). Taxonomic classifications are presented at the most fundamental levels of taxonomy (see Table S2). [file EMI4-16-e13228-s004.jpg]
